# Perspectives of underweight people with eating disorders on receiving Imagery Rescripting trauma treatment: a qualitative study of their experiences

**DOI:** 10.1186/s40337-022-00712-9

**Published:** 2022-11-30

**Authors:** Marieke C. ten Napel-Schutz, Simona Karbouniaris, Suzanne H. W. Mares, Arnoud Arntz, Tineke A. Abma

**Affiliations:** 1 Department of Eating Disorders (Amarum), GGNet Mental Health, Warnsveld, The Netherlands; 2 Radboud Centre Social Sciences, Radboud University, Nijmegen, The Netherlands; 3 Utrecht University of Applied Sciences, Utrecht, The Netherlands; 4 Leiden University Medical Centre, Leiden, The Netherlands; 5 Department of Clinical Psychology, University of Amsterdam, Amsterdam, The Netherlands; 6 Department of Public Health and Primary Care, Leiden University Medical Centre, Leyden Academy on Vitality and Ageing, Leiden, The Netherlands

**Keywords:** Eating disorders, Underweight, Anorexia nervosa, PTSD, Trauma treatment, ImRs, Qualitative research

## Abstract

**Background:**

The prognosis for underweight individuals with an eating disorder (ED) and posttraumatic stress disorder (PTSD) is worse than for their peers without these comorbid symptoms. This qualitative study explores the experiences of trauma-focused Imagery Rescripting (ImRs) therapy of underweight inpatients being treated for an ED.

**Objective:**

To test the feasibility and to improve ImRs by understanding the experiences and perspectives of people with an ED and PTSD who, when underweight, received ImRs as an adjunct to their inpatient ED treatment.

**Method:**

To explore how underweight people with an ED experience and perceive ImRs, we used a qualitative study design involving semi-structured interviews with 12 participants. After analysis, the data were summarized and classified within a thematic framework that focused on experiences and improving the ImRs method.

**Results:**

The thematic analysis resulted in the following 6 main themes; (1) Expectations of ImRs; (2) Ability to participate in ImRs; (3) Effect of ImRs; (4) Experience of ImRs technique; (5) Conditions under which ImRs is given; (6) In depth-analysis. The results show that despite the fear of disappointment the participants appreciate addressing the PTSD and ED symptoms simultaneously. Further, results showed that it had been possible for them to attend ImRs but that the effects of ImRs were not uniformly perceived. Also, participants indicated that a caring context is important and that ImRs should not be scheduled immediately before a meal. Finally, the treatment generated hope.

**Conclusions:**

The findings of this study demonstrated the feasibility of the integration of ImRs trauma treatment for individuals who are being treated in an ED inpatient treatment setting, and are in contrast to standard practice where the focus of inpatient treatment has been ED-symptom improvement without comprehensively addressing past traumatic experiences during an underweight phase.

*Trial registration* International Clinical Trials Registry Platform (ICTRP) (NTR6094). Date of registration 09/23/2016. https://trialsearch.who.int/Trial2.aspx?TrialID=NTR6094

**Supplementary Information:**

The online version contains supplementary material available at 10.1186/s40337-022-00712-9.

## Plain English summary

A proportion of those with eating disorders have also experienced traumatic events and ongoing symptoms of PTSD such as re-experiencing of the trauma and nightmares. We implemented an innovative trauma intervention called Imagery Rescripting (ImRs) to explore whether for those undergoing inpatient treatment for an eating disorder (in an underweight phase), it would be possible to treat the various trauma-related symptoms as well as the eating problems. Since this has not been investigated before, we asked the participants in this study to recount their experiences. Twelve participants who were underweight, reported a past history of trauma and were in an inpatient eating disorder treatment program participated in ImRs therapy intervention. One of these participant did not engage in the ImRs therapy because she discontinued the inpatient ED treatment. Analysis of interviews with these participants found that -although they were reluctant before the start of the treatment- the ImRs treatment during their inpatient admission had given them hope again. They added that it was important to have support from group members, sociotherapists and therapists. They shared a number of ways that the ImRs treatment could be adapted to people with eating disorders. Their experiences indicated that given these factors it was possible to treat PTSD during an underweight phase. This is important: until now, treatment for eating disorders has not specifically been trauma-focused and these tips have scope to improve the ImRs intervention and eating disorder treatment more broadly in the future.

**Supplementary Information:**

The online version contains supplementary material available at 10.1186/s40337-022-00712-9.

## Background

Post-traumatic stress disorder (PTSD) symptomatology is a specific risk factor for the development of an eating disorder (ED) such as Anorexia Nervosa (AN) [1]. Researchers have hypothesized that certain ED behaviours may help a person to reduce, avoid, or escape from PTSD symptoms [1, 2, 3]. Between 10 and 47% [4, 5] of people with AN are diagnosed with PTSD—a group, particularly the patients treated in higher levels of care, who are known not only to be harder to treat than those without PTSD [3, 6, 7], but also to have a greater number of other co-morbid symptoms (such as anxiety and depression symptoms, obsessive–compulsive symptoms, and interpersonal symptoms), and also lower self-esteem and poorer quality of life [2, 6, 8, 9, 10].

It is therefore unsurprising that several studies have shown that this group of people respond worse to treatments, quit treatment prematurely, or are more likely to relapse, than people who have AN without PTSD [2, 3]. Patient organizations and several researchers have therefore long proposed that treatment outcome might be improved by treating trauma at an earlier stage, i.e. during an underweight phase (IxtaNoa, personal communication, May 2012) [2, 3]). Some studies have been done on treating trauma symptoms in addition to ED symptoms [9, 11, 12]. But very little research has investigated the options for treating trauma symptoms during an underweight phase.

In the clinical tradition to date, psychotherapeutic trauma-focused treatment is not initiated in people who are underweight [13, 14]. This is because it is assumed that, during periods of underweight, cognitive functioning is suppressed [15, 16, 17], too few emotions are experienced [18], and emotion regulation is poor [19, 20]. But although poor functioning in these areas is assumed to obstruct effective trauma-focused treatment, there is no research consensus on poor cognitive functioning, failure to experience emotions, and the associations between body mass index (BMI) and emotion-regulation skills.

To seek a solution to the lack of evidence and the need for appropriate and helpful treatment, a broader study by ten Napel-Schutz et al. [21] incorporated a quantitative multiple baseline case series study involving ten participants with PTSD, an ED, and a BMI of between 14 and 16.5. The results of the latter showed that people with PTSD could be treated effectively when underweight. The following symptoms were all reduced: PTSD symptoms,emotional experiences of disgust, shame, guilt and anger; negative cognitions about the body, the self and the world; and difficulties with emotion regulation. The eating pathology also became less severe [21].

The treatment method used in this case series study was the evidence-based treatment ImRs for PTSD (Additional file 1: box S1) [22, 23, 24, 25]. Although ImRs had not yet been used with people who were underweight, it was used here because earlier studies had shown (1) that another trauma method (EMDR) requires sufficient physical and mental stability because it can be an invasive procedure [26], (2) that ImRs refrain from full exposure to traumatic memories and therefore may present fewer difficulties than treatments that do rely on full exposure [27], (3) that participants in the study of [28] found ImRs to be less invasive than exposure, and (4) that here was less dropout with ImR than with pure Imagery Exposure and this was seen as an indication of the less invasive experience of ImRs [29], and (5) that it achieved good results with perceived anger, guilt, and shame [28].

Because ImRs had not previously been offered for PTSD during an underweight state and during clinical admission, it is helpful to know what kind of experiences participants have, how they perceive the treatment, and what, from their perspective, can be done to improve it in ways that will better attune it to their needs. To gain insight into the experiences of trauma treatment with ImRs in people being treated in an inpatient ED setting, the present qualitative study therefore aimed to explore individuals’: (1) Expectations of the treatment; (2) experiences of IMRs treatment, including any perceived shifts in cognitive functioning, emotional experiences and their regulation; and (3) perspectives on the feasibility of treating trauma when they were also underweight.

## Method

### Setting

Parallel to the inpatient treatment they were receiving for their ED while underweight, participants in the present study received twelve ImRs sessions. All the participants had been diagnosed with PTSD and were female; their demographic and clinical data are listed in Additional file 1: Table S1. The interviews were held two weeks after the end of ImRs treatment, at that time, 1 participant had already stopped inpatient ED treatment while the other participants were still receiving it.

### Ethics

Approval for the intervention study, and thus also for our qualitative study, was obtained from the medical ethical committee at the University of Amsterdam (2016-CP-7111). Informed consent was signed by all participants. Pseudonymity was guaranteed, and was achieved by deleting the participants’ names and personal characteristics from the data, and by not sharing the transcripts with the therapists and other peers who had inpatient treatment for their ED. All statements in this article have been disconnected from the people who made them. The verbatim transcripts and recordings will be destroyed five years after publication of this article. Although, conceivably, participants may be able to link some statements to fellow participants, all committed themselves to confidentiality agreements at the start of clinical treatment.

All participants were told that it was possible and permissible not to answer questions. The researcher/first author is a clinical psychologist who works at the same institute but had no therapeutic relationship with the participants. Before agreeing to participate, participants had received an announcement and an explanation of the study, accompanied by an invitation to participate.

### Design

As we wished to improve the treatment by learning more about the participants’ personal experiences and perceptions, and also by knowing what meaning they would attribute to ImRs, we chose a qualitative study with interviews. We chose a semi-structured approach that was more descriptive than phenomenological or interpretive, as this would be better suited to our aim of eliciting information that would help to improve the treatment.

### Data collection

Data collection involved semi-structured interviews with participants [30]. These were based on a pre-defined topic list, whose initial draft was first discussed with an ImRs professional, with whom it was then confirmed. The validity of the topic list was then checked in three individual interviews with participants. After the addition of several topics, the definitive topic list was then established (see Additional file 1: Table S2).

Each interview started with an introduction to the interview and with questions on sociodemographic data, after which the topics on the topic list were discussed (see Additional file 1: Table S2).

The interviews were held in a therapy room at the institute where the ED treatment took place. Each interview was led, guided and structured by the same person (the first author), who ensured that all topics were discussed. She also took fieldnotes. To make sure all topics were addressed and the answers were properly understood, she asked questions and listened actively, connected information in her fieldnotes, and, in collaboration with the participants, she tried to understand as best as possible what the participant meant [30]. The interviewer was as open as possible to the experiences of the interviewee, an intention that was emphasized during the interview. Care was taken to prevent common pitfalls, such as outside interruptions, stage fright, awkward questions, jumping from one subject to the other, and the temptation to counsel respondents [30].

All interviews were recorded on an mp3 player after the participants’ approval had been received. They were elaborated verbatim. A summary was written afterwards. The interviews had no fixed duration, but ended when all topics had been addressed and no new information emerged. Their length ranged from 30 to 90 min.

### Sampling

We used an iterative process in which the topic list was adjusted after the first three interviews. In qualitative research it is customary to select a relatively small group of respondents until data saturation is attained [30]. As most interviews achieved saturation on most of the predefined and some extra topics, it was possible to make valid statements on these topics. However, some topics were so person-related that saturation was not achieved. The repeated themes create the opportunity to gain a thorough understanding of experiences [31]. Twelve people were invited for the interview: the ten completers of the ImRs treatment, one person who had dropped out of treatment (after three weeks of ImRs, and one person who did not start ImRs because she could not sustain the clinical ED treatment. She discontinued clinical ED treatment before the start of ImRs because she had difficulty with participating in the ED treatment group,she was diagnosed with an autism spectrum disorder. She was interviewed to find out if the upcoming ImRs treatment was a reason for her to quit. One completer, had hallucinations in addition to PTSD that were trauma related and had no bizarre content. These secondary psychotic symptoms were not considered contraindications for participation in the ImRs. The hallucinations did not get worse but also did not diminish after the first ImRs sessions so anti-psychotic medication was given for a short period of time without result. The patient herself was very eager to complete the ImRs so she was included in the results as a completer.

### Analysis

In line with the framework analysis developed by the National Centre for Social Research,^1^ we summarized and classified the data within the following thematic framework: (1) The researcher familiarized herself with the data by listening to the recordings and re-reading the fieldnotes and transcripts; (2) A framework (coding scheme) was developed, based partly on the themes that guided the interviews, and partly on the issues that arose in the transcripts; (3) The text fragments in the transcripts were coded and labelled using the coding scheme; (4) Indexing was done intersubjectively by two researchers, who worked systematically on themes and sub-themes; (5) The associations between these themes and sub-themes were then established; and (6) Working independently, the researchers coded and categorized the themes and sub-themes they contained. If disagreements arose on coding, they were discussed until consensus was reached. With the support of the qualitative data-analysis program atlas-*ti*, the data was charted, i.e. rearranged by theme*.*

It was particularly useful that the second researcher not only had a research background but was also an expert-by-experience: previous research has shown that such a dual identity can be advantageous. When a study is conducted only by professionals, it is easier for sight to be lost of the patient’s perspective [32, 33, 34].

### Quality procedure

As a validity check we used respondent validation, in which each participant was sent the verbatim transcript and a short analytical summary of their interview by secured email [30, 35, 36]. Participants were asked if the interpretations corresponded with their perspective, and if they expressed what they had meant. This respondent validation showed that all participants agreed that the summary of their interview was accurate. One participant commented that during the interview she looked back positively on the ImRs treatment, but that her opinion had changed by the time respondent validation took place. She indicated that she had not managed to (1) Indicate that conditions at the time of ImRs were not optimal, and (2) that she now felt that she had needed longer/extended ImRs treatment.

### Trauma-focused ImRs

Participants were offered twelve 90-min individual ImRs trauma sessions over six-weeks [25]. ImRs is a technique that attempts to change the meaning of an experience by looking at traumatic childhood memories in a different way. This experiential therapeutic technique uses imagery and imagination to imaginatively intervene in traumatic memories. The trauma memory is activated, i.e. the patient imagines the start of the traumatic memory and when there is enough emotional activation, the rescripting starts. The therapist (first six sessions), and in the last six sessions the participant (each from their current perspective), then rescripts the traumatic experience in a more desirable direction, while imagining this new script in the liveliest way possible. For a more detailed treatment description see Additional file 1: box S1. Four participants experienced physical assault, 5 participants experienced sexual assault, 2 participants had other unwanted or uncomfortable sexual experiences, 1 participant experienced combat or exposure to a war zone in the military and 1 participant experienced another very stressful event. One participant experienced 2 one-time traumatic experiences, the other participants experienced more complex trauma related to prolonged and severe traumatization at a younger age [21].

### ED treatment

In the clinic the participants followed a programme for their ED that consisted of inpatient treatment five days a week. They spent their weekends at home. The programme used cognitive behaviour-change methods and focused on weight gain and on the factors that were maintaining the ED. When they were underweight, participants committed to a weekly weight gain of 700 g. The therapeutic programme was offered entirely in group form and consisted of (1) three supervised main meals and snacks daily, (2) cognitive behavioural therapy (focused on the thoughts and behaviours associated with the ED), (3) eating-behaviour therapy (focused on the eating diary), (4) body and movement therapy, (5) psychoeducation, (6) progress and goal discussion, (7) psychodynamic group therapy (addressed the factors that caused and/or perpetuated the psychological problems and focused on both the individuals in the group and the interaction between group members), and (8) occupational therapy. Additionally, five times during their stay, participants were given opportunities to take part in a parent-and-partner group.

## Results

The topic-list questions produced five fixed themes. Close reading then produced a further 25 sub-themes. Through an iterative process, an additional theme with 3 sub-themes was added. The 28 sub-themes describe a total of 81 categories.

Below, the results are described on the basis of the six themes and 27 sub-themes. Additional file 1: Table S3 provides an overview of the themes, subthemes, categories and illustrative quotes. Additional file 1: Table S4 provides an overview of tips and suggestions from a participants' perspective.

### Expectations

Overall, all participants had appreciated the opportunity to participate in this study, particularly because of the opportunity to address the symptoms of the ED and the PTSD simultaneously while they were underweight. Many had gone through a long search for adequate treatment. *"I was very happy there was a clinic somewhere that was willing to offer trauma treatment even though I was underweight."* Although three participants had had positive expectations of ImRs, the others had been coloured by earlier negative experiences of EMDR treatment, fearing disappointment, no longer dared to expect anything and had chosen to take the trauma treatment without expectations.

As well as indicating that it was difficult, a priori, to get a good idea of ImRs, two participants had also had doubts about its effectiveness: *"Because the therapist explained that you can make up all sorts of things in your imagination, I wondered something like ‘is this going to work on my trauma…?’."*

With regard to their preparation for the study, all participants indicated that they had received sufficient information. (See Tips and Suggestions Additional file 1: Table S4, point 4.1; Illustrative Quotes, Additional file 1: Table S3, points 1.1 and 1.2.)

### Ability to participate in ImRs

In the interviews, participants talked about the ability to participate in ImRs in relation to their level of concentration, their ability to experience feelings, their ability to regulate emotions, gaining weight, their ability to imagine, their physical effects, their trauma topicality and openness about their trauma.

#### Ability to control attention and to concentrate during ImRs

With one exception, all participants indicated that their attention span had been sufficient to do the ImRs, even though they were underweight and had other co-morbid disorders (Attention Deficit Hyperactivity Disorder, Tourette Syndrome etc.). *“Yes, I was generally able to concentrate sufficiently.”* The exception indicated that, after a full day of therapy, she had been too tired for the ImRs treatment. Although she dropped out after three sessions of ImRs, she had experienced a positive result on her PTSD symptoms, but had been too tired the next day to follow the clinical treatment properly. *“I managed, but it was such an effort that on the following day I could no longer concentrate on the therapy in the clinic.”*

Ten participants reported adequate concentration. Of these, six participants indicated that three factors had made it difficult to maintain their concentration throughout the ImRs session (90 min): the time of day, the length of the ImRs session, and the many distractions caused by ambient noise. “*You hear things in the hallway, so it’s difficult to maintain your concentration.”* The therapists found solutions to these distractions: a quiet room, turning off the light, shortening the sessions, and finding a more suitable time for the ImRs. Despite the difficulties, three participants reported that well-coordinated individual person-oriented support had eventually made it possible for them to concentrate properly. (See Illustrative Quotes, Additional file 1: Table S3, point 2.1; Tips and Suggestions, Additional file 1: Table S4, point 4.2.)

#### Ability to feel

The interviews showed clearly that almost all (10) participants experienced enough emotions to do ImRs: *"Yes, I’m still a bit overwhelmed: I could feel them very well."* One person said she had tried to avoid her feelings, but her therapist saw from her body signals that the tension was building up. Especially at the end of the sessions, after ImRs, she had a lot of feelings*.*

In the period in which ImRs was provided, two participants experienced a change in their ability to feel, even when their body weight increased. *"No, I don't really feel as if it changed very much."* Although one participant indicated that she had been able to access her feelings better during the second six sessions, she attributed this to the method. *"From the moment I started to intervene myself it became a bit easier."* (Illustrative Quotes, Additional file 1: Table S3, point 2.2; Tips and Suggestions, Additional file 1: Table S4, point 4.2.)

#### Ability to regulate emotions

Seven participants indicated that they found it scary to feel emotions they had not felt for an extended period or had done their best to avoid. One participant said: *"I feel everything again, and that's just what I wanted to get rid of."* Another: *"I'm not used to feeling emotions at all any more—I've always pushed them away, and it's scary when they’re there again."*

During ImRs they were asked to start feeling these emotions. As this differed slightly from the ED treatment, in which they tried to let go of their old coping strategy of regulating emotions through ED behavior and thoughts, it evoked not only the fear of getting overwhelmed and lingering in the emotion, but also the experience of managing not to be flooded by emotions. *"And then I was crying really hard and then it was okay too—not that I got stuck in it, because that was what I was really afraid of: getting stuck in it."* They indicated that ImRs helped them more than previous methods to learn to regulate their emotions. They also reported that it helped that they could do ImRs in a safe clinical treatment environment where emotions were welcomed, and where they could cry, talk with peers and sociotherapists; or just go for a walk. (Illustrative Quotes, Additional file 1: Table S3, point 2.3; Tips and Suggestions, Additional file 1: Table S4, point 4.2.)

#### Ability to gain weight, and the role of weight and underweight

Participants had different experiences of being able to gain 700 g body weight per week during ImRs. While two participants said that the trauma treatment required too much energy for them to be able to continue the growth curve, all the others were able to manage both. However, as their trauma symptoms seemed to become worse or more noticeable when they gained weight, these participants were unanimous that help was needed to continue to put on weight while simultaneously handling the trauma symptoms. *"Yes, [weight gain] did make me feel worse, and I really felt that you shouldn't wait to help me until I'm at a healthy weight, as that would make it very difficult for me actually to attain that weight.”* For this reason, participants experienced the support they received at mealtimes during the ImRs period as essential to gaining or maintaining a stable weight during the ImRs treatment. *"So, yeah: seeing I've previously had trauma treatment that gave no support to eating—where I actually went on hunger strike—yeah, I'm glad [ImRs] was done in a clinical setting."* (Illustrative Quotes, Additional file 1: Table S3, point 2.4; Tips and Suggestions, Additional file 1: Table S4; 4.2.)

#### Ability to imagine

Many individual differences emerged regarding participants’ ability to work with imagination. Four could easily imagine situations and even marvelled at it*.* One participant noted that she was a visual thinker, so this technique suited her very well: *"I was surprised how well I could imagine it."* Two participants had to get used to it, after which it worked well. Two participants mentioned that, if you are an image thinker, the pictures really come into your mind, are extra vivid, and that if you think more rationally, it can be hard to imagine things that are not realistic or have not happened. For one person, imaging the therapist in the image did not work, as it was too far removed from reality. It had then helped her that she and her therapist decided to adapt the script and stay closer to reality. (Illustrative Quotes, Additional file 1: Table S3, point 2.5; Tips and Suggestions, Additional file 1: Table S4; 4.2.)

#### Ability to engage in ImRs: physical effects

Four participants found that ImRs caused many physical reactions, such as headaches, sweating, palpitations, fatigue, pain in the legs, paralysis of limbs, and an inability to move the legs. *"I had a lot of physical reactions during and after ImRs—it was quite intense."* Although one participant stopped ImRs due to fatigue, the physical reactions were not a reason for the other participants to discontinue it. Someone verbalized that the physical reactions decreased because they had learned to reassure themselves.

The participants with physical reactions indicated the importance of announcing in advance that multiple physical reactions could occur. As well as indicating the importance of the therapist remaining calm and looking for person-oriented entrances, they made several suggestions for making it possible for a participant to continue ImRs treatment despite the physical reactions; in the event of limb failure, for example, it helped to stand during the session. It also helped to have a wheelchair ready, not to schedule the ImRs sessions two days in a row, and to make it possible for participants to remain in the room after a session until they felt grounded again.

In summary, although participants experienced many physical reactions, these were a reason for hardly any of them to stop ImRs. (Illustrative Quotes, Additional file 1: Table S3, point 2.6; Tips and Suggestions, Additional file 1: Table S4, point 4.2).

#### Ability to engage in ImRs: trauma topicality and openness about trauma

Regarding the topicality of the trauma—in other words, the extent to which they were still troubled by it—it emerged that two participants were not yet fully in a safe environment outside the clinic (i.e. in their private lives). Three participants indicated that their traumas were no longer topical but that tensions could still be evoked, as they still had contacts with the perpetrator. For example, one perpetrator occasionally visited over the weekend; another continued to send messages; and as one group of perpetrators still lived in the same area, the participant occasionally ran into them.

Two participants indicated that, with a therapist they knew barely or not at all, they had difficulty being open about the trauma they had experienced. They sometimes found it difficult to share at all. One person indicated that she had started to write down her whole trauma story not only for the therapist to read, but also for any future therapists; this would make it unnecessary to tell her story again. Two participants indicated that they were unable to tell the whole story about the events, one reason being that they did not dare to complete certain memories in their mind. Another participant gradually increased her degree of openness, starting with the less difficult trauma images, and eventually discussing the more difficult images only in the final ImRs sessions.

Three participants indicated that the more often there was openness, the easier it went. If it was possible to be completely open, this was experienced as pleasant. But overall, all participants developed sufficient trust during the sessions to provide enough disclosure about their traumas. (Illustrative Quotes, Additional file 1: Table S3, point 2.7; Tips and Suggestions, Additional file 1: Table S4, point 4.2.)

In summary, participants indicated that, given well-coordinated person-oriented adjustments and a joint search for ways to maintain the treatment with the clinical treatment team and therapists, the following had been possible: to attend the treatment, to apply the imagining technique, to tolerate the physical effects, and to disclose their traumas sufficiently. They could do this with sufficient concentration, emotional experiences, and emotion regulation; and they were able to maintain or gain weight.

### Perceived effect of ImRs

In the interviews, participants talked about their perceived effects of ImRs in terms of negative emotions, eating disorder symptoms, PTSD symptoms, self-compassion, internalization of ImRs and expected effects of ImRs in the future.

#### Perceived effect of ImRs on negative emotions

Although two participants found it difficult to feel anger, two participants noted that this improved during the ImRs sessions. Three participants indicated that they started to feel angrier about what had been done to them. Another participant noted that she succeeded in feeling less angry, and two participants in directing their anger less towards themselves but also towards the perpetrator or perpetrators. *"First I was only angry at myself, and now I’m also angry at the people who did that to me."* It emerged that six participants had been able to re-frame the trauma. (Illustrative Quotes, Additional file 1: Table S3, point 3.1.)

Different experiences emerged regarding the effect of ImRs on guilt. Four participants started to feel less guilty or were able to reframe the traumatic experience. *"What happened really shouldn't have happened, and I was able to experience that it had not been my fault."* Two participants noticed no difference in their feelings of guilt. However, there was also someone (who also experienced psychotic symptoms) who started to feel more guilty. *"If only it had happened like that, if only I had made a different choice."* (Illustrative Quotes, Additional file 1: Table S3, point 3.2).

With regard to the perceived effect of ImRs on shame, two participants reported that shame stayed out of the picture during treatment, and, even though the qualitative questionnaires had explicit questions on it, that the therapist had not asked about it directly. Typically, shame concerned questions of what the participants would have liked to do with the perpetrator or perpetrators, or shame about their own physical reactions to ImRs. *"If you have so much shame, you’re not going to talk about it."* It was suggested that the therapist should ask explicitly about feelings of shame. *“If we’d been asked, I think it would have come up more often. Otherwise, if you’re so ashamed, you’re not going to tell them.”* It also helped two participants that they could do whatever they wanted to do in their thoughts, and that they did not have to speak out loud. The therapist could support this process by taking a robust approach to it, by keeping calm, and by agreeing with participants what they should include in the imaging in order to reduce the feelings of shame. (Illustrative Quotes, Additional file 1: Table S3, point 3.7; Tips and Suggestions, Additional file 1: Table S4, point 4.3.)

#### Perceived effect of ImRs on ED symptoms

With regard to the effect of ImRs on eating, eight participants stated that, due to the emotions they had experienced through ImRs, it had been difficult to eat a meal after the session on the day of the ImRs. After ImRs, participants typically reported “*feeling dirty*”, having “*too much tension*”, having “*too many sensory re-experiences”*, and having “*food triggers*”; or “*not being hungry when very emotional*,” and “*not being able to eat because of a lump in my throat.*" One participant reported difficulties also the day after ImRs. Four participants also indicated that they used "not eating" on the day of ImRs to avoid or distract themselves from emotions such as sadness and anger, and that the ED helped to push the emotions away. *"ImRs makes it hard to get through the day. So, to suppress those feelings a bit again, I did tend to cut down on food."* (Illustrative Quotes, Additional file 1: Table S3, point 3.3).

It is noteworthy that the topic of negative body perception was not addressed very much during the ImRs sessions. As one participant linked her negative body awareness to the ED and not to the trauma, she had no hope of change through ImRs. However, another participant who did discuss her negative body awareness during ImRs also experienced no effect of this on her body awareness: “*My body still feels dirty and all that.”* Two participants indicated the importance of explicitly discussing any negative body perceptions that may have originated from the traumatic event. (Illustrative Quotes, Additional file 1: Table S3, point 3.4; Tips and Suggestions, Additional file 1: Table S4, point 4.3).

#### Perceived effect of ImRs on PTSD symptoms

Participants reported variously that ImRs had had positive, neutral, and negative experiences on their PTSD symptoms.

With regard to its positive effect on PTSD symptoms, someone expressed surprise at how easily the brain could be changed. In her case, when she thought back to the unpleasant event, she now saw the therapist in the picture. Three participants stated that they had managed to stay in the here and now better by thinking about the rescripted situation. *"Now I can also say to myself: it’s not now, it was then. And then I try to see a bit of the rescripted scene in front of me. So it will end well."* Three participants also reported that their reactions to re-experiencing symptoms became less intense after a few ImRs sessions, and that their physical reactions, such as palpitations and sweating, and their nightmares, flashbacks, and emotional reactions to ImRs, had decreased. *"No more crying all the time.”*

One participant indicated that she had noticed little effect on her PTSD symptoms. She did not feel that she was thinking about her trauma less, or was less sensitive to stimuli, and still felt very vulnerable, unstable, and emotional. *“No, I don't think anything has changed regarding the degree of those symptoms.”*

Two participants reported a temporary aggravation of their PTSD symptoms, consisting of an increase in re-experiences, some of which became more intense during the period or on the day of the ImRs. During a re-experience they did not manage to go back to what had been done in the rescripting. They indicated that when a re-experience began, they consciously tried to call up the image from the session. They could then see this new image for a moment, after which the old image took over. *" Sometimes, when images start coming up, I consciously try to call up an image from those sessions. But often I see it just for a moment and then it’s gone again—the other image, the real one, flies over and covers it."* Three participants had a re-experience or dissociation during the ImRs sessions. *"During ImRs I often found myself going into a kind of express train, so to speak, and the film started running for me, so I was actually in a re-experience I couldn’t escape from.”* After the sessions, two participants suffered a lot from nightmares.*" And certainly, in the nights after the sessions I also had a lot of nightmares."* (Illustrative Quotes, Additional file 1: Table S3, point 3.5).

Five participants indicated that ImRs initiated a grieving process whereby, through rescripting, they realized what protection or care they had missed at the time of the traumatic event. *“[It’s a] process of mourning… and you think…'if only I’d had that.’”* (Illustrative Quotes, Additional file 1: Table S3, point 3.6).

#### Perceived effect of ImRs on self-compassion, internalization and its expected future effect

The treatment was also found to influence the development of self-compassion. Self-compassion seemed to have increased in three participants, though a few (2) did not experience this. For example, while one participant did not at first know what to say to reassure herself, her ability to do so improved during the session. In contrast, two other participants indicated that they had not learned to look at themselves with more compassion. (Illustrative Quotes, Additional file 1: Table S3, point 3.10).

At the time of the interviews, it seemed that seven participants had not yet (fully) internalized the ImRs method, and were unsure whether they could expect any effect from it over time. However, two participants were already able to use it independently. (Illustrative Quotes Additional file 1: Table S3, point 3.5).

When asked if they expected any future effect from the ImRs treatment, participants had various ideas. Five participants indicated that they did not know: “*I’ve no idea what to expect.”* Two participants thought it would have not any further effect, while four participants thought the effect they had experienced would develop further: *“I hope so, and definitely think it will do something.”* They added that while it was the start of a nice change, it was not enough yet: *“I don't know if this will get rid of it completely, but I do think it will do something.”* (Illustrative Quotes, Additional file 1: Table S3, point 3.11).

### Experiences with the ImRs technique

In the interviews, participants discussed their experience with the ImRs technique imagination with rescripting and their experiences with this technique in the first and second six ImRs sessions.

#### Imagination and rescripting

Seven participants reported that the imagining had gone well and were amazed how well they had been able to experience it. “*Surprised how well I could imagine it and how it felt exactly like the time of the trauma*.” Five of the participants also reported that they liked the fact that they could keep control. “*I found it quite tough, but also a very nice treatment, as you get control and keep it.”* One participant said that when she realized that she herself could determine the intensity of the tension she felt, she started to dread it less. *“I was not looking forward to it, and after the first session I was dreading it even more. And after that it got less, because at a certain point I realized that I could determine how great I allowed the tension to become—to what extent I went back into that memory. I liked that.”*

On the other hand, two participants indicated that the method made it possible to avoid unpleasant feelings or images. For example, one participant indicated that there were unfinished images to which she did not dare to go. During the rescripting, she did not have to finish these images. (Illustrative Quotes, Additional file 1: Table S3; 4.1, Tips and Suggestions, Additional file 1: Table S4; 4.1.)

#### The first six sessions

In the first six sessions, the therapist steps into the image and intervenes in coordination with the patient. Two participants reported that this was difficult in the beginning, as they had never shared certain events before. Seven participants indicated that it was nice that the therapist had intervened during the first six sessions, that they had not had to do it alone, and that there had been an example: *"When X did it, I was pleased that she acted a bit more angrily than I could.”* But at the same time, two people also found it confronting that no-one had helped in the past.

Five participants felt that rescripting with the use of fantasy felt unrealistic and rather crazy, which also could limit the imaging. They were aware during rescripting that the rewritten situation did not correspond with reality. One person became aware, especially after the rescripting, that if a rewritten and imagined situation was a bit bizarre, they sometimes experienced it as unrealistic. However, another participant found it impossible to let the therapist enter the picture because it was too unrealistic for her, which is why she imagined the help from her parents.

During rescripting, participants were asked what their needs were. Two participants experienced the question of need to be difficult or confronting, but two participants experienced the questions also as a question that was very nice. *"I found it quite difficult, as I very often didn’t know what my need was. But I liked the fact that she had asked, because the question helped me realise much more strongly that I could think of something I actually wanted."* However, two participants also stated that identifying the need became easier along the way. *"As the sessions progressed, I was increasingly able to communicate what I needed*.” (Illustrative Quotes, Additional file 1: Table S3, point 4.2; Tips and Suggestions, Additional file 1: Table S4, point 4.4.)

#### The last six sessions

In the last six sessions, the participant rescripts herself. Here the experience was mixed. Five participants found these sessions to be nicer than the ones guided by the therapist: they felt more empowered, were more in control, and could create their own narrative. *"I liked the second part better because I could do it myself. And after that I felt more powerful.”* On the other hand, three participants found it more difficult to take the lead. Sometimes, for example, the connection with the child in the image was completely broken. In two other cases, they did not dare to speak to the perpetrator out loud, failed in their attempts to comfort the child, or found it unpleasant to have to do it alone. *" Having to step into the picture myself, as an adult—that took a lot of effort, especially because I noticed that I did not see my little self as myself. As if I were helping another child.”* At the end of each ImRs sessions, the therapist asked participants to bring their attention back to the present. Two participants reported that this was not an easy process, and that they still felt “floaty” for a while*.* (Illustrative Quotes, Additional file 1: Table S3, point 4.3; Tips and Suggestions, Additional file 1: Table S4, point 4.4.)

### Contextual conditions

The ImRs was offered at the time of inpatient admission for the ED treatment. In the interviews, the participants shared their experiences with the support in the clinical setting, with treating multiple participants at the same time, with beliefs about ImRs in a clinical setting, with the support during meals, about the combination of ImRs in a clinical setting and about the number, the timing and the scheduling of the ImRs sessions.

#### Clinical setting; support

Nine participants particularly appreciated ImRs treatment in the context of the ED facilities. They gave several reasons for this, such as the distraction, the structure, the relaxation, and the fact that it felt too heavy to return home after ImRs. The support after ImRs was particularly appreciated, as it had made it possible to talk to the sociotherapists, group members, or roommates; or to go for a walk, to avoid getting stuck in unpleasant feelings, or to prevent withdrawing. *"And they [the sociotherapists] also helped me with re-experiences or dissociations, so to speak—they helped me through it. And after that they made sure I was completely here again and safe.”* However, one person indicated that she needed more support, as she had mostly coped alone. She had found it difficult to ask for support. There were also two participants who just wanted to be alone for a while after ImRs and had no specific need for support. (Illustrative Quotes, Additional file 1: Table S3, point 5.1; Tips and Suggestions, Additional file 1: Table S4, point 4.5.)

#### Clinical setting; several participants at the same time

It happened twice during the study that two participants in the same ED treatment group received ImRs in the same period. One participant reported that a fellow patient’s re-experiences had triggered her own re-experiences. Two participants indicated that, at times, they felt guilty when they needed the sociotherapists’ support at the same time as someone else, as this prevented the rest of the clinical group from getting attention (Illustrative Quotes, Additional file 1: Table S3, point 5.1.2; Tips and Suggestions, Additional file 1: Table S4, point 4.5).

#### Clinical setting; belief about ImRs

Two participants indicated that at times it seemed that participants and clinic staff had the idea or belief that undergoing ImRs treatment was very intense. A participant who did *not* think it was so intense therefore became uncertain about whether she was entitled to ImRs because she did not experience it like that (Illustrative Quotes, Additional file 1: Table S3, point 5.1.3; Tips and Suggestions, Additional file 1: Table S4, point 4.5).

#### Clinical setting; mealtime support

By offering ImRs in the context of the ED facility, there was active support around meals. Almost everyone (9) found this to be supportive. *"If I hadn't been in this environment, I think I would just have stopped eating."* (Illustrative Quotes, Additional file 1: Table S3, point 5.1.4; Tips and Suggestions, Additional file 1: Table S4, point 4.5).

#### Clinical setting in combination with ImRs

Two participants found it difficult to undergo a six-week programme that combined two weekly 90-min ImRs sessions with the inpatient ED-treatment programme. *"Found it quite tough to have one of those sessions twice a week in the inpatient programme—that really took a lot of energy out of me, so yes, I did find it tough."* Five participants who had managed this full programme were positive about the fact there had been sufficient closure time after the rescripting. The participant who stopped after three ImRs sessions indicated that it had been too hard for her to attend ImRs as well as the clinical ED programme: *"Unfortunately that was just a bit too much—I did benefit from it, but I was so tired that I no longer had the energy to continue.”* (Illustrative Quotes, Additional file 1: Table S3, point 5.2.1; Tips and Suggestions, Additional file 1: Table S4, point 4.5.)

#### Number of ImRs sessions

Opinions about the number of ImRs sessions differed widely. While some participants thought that 12 sessions had not been enough, others thought 12 was enough or even too many. Three participants who had experienced multiple traumas, however, indicated that 12 sessions may have been too few: “*I had quite a lot of memories I wanted to discuss, [and] didn't get to do them all. I needed more than 12 sessions.”* (Illustrative Quotes, Additional file 1: Table S3, point 5.2.2; Tips and Suggestions, Additional file 1: Table S4, point 4.5.)

#### Timing of ImRs

A six-week naturalistic baseline had been followed by a random baseline period. Six participants felt that the start of ImRs had been well timed, because they needed some time to get used to the ED treatment. *"Just a few weeks to get used to the programme in the group”.* Two participants also indicated that if their weight had been lower than the weight specified in the study’s inclusion criteria, ImRs might not have been successful. *"If my weight had been lower, ImRs probably wouldn’t have been possible. I was in a phase of not feeling. If I’d had to start feeling then, I don’t know if I could have, and I wouldn't have dared to make the step towards."* One participant indicated that, for her, the onset of the ImRs treatment should have been earlier. She had taken a pause from the clinical programme two weeks before starting ImRs because the PTSD symptoms became too severe. *"I didn't like it because the trauma symptoms had become so severe, and nothing was being done about them yet. The only thing being stressed [i.e., during the ED treatment] was eating. While eating had become extremely difficult because of all the re-experiences.”* (Illustrative Quotes, Additional file 1: Table S3, point 5.2.3; Tips and Suggestions, Additional file 1: Table S4, point 4.5.)

#### Scheduling of ImRs

In principle, the ImRs sessions were scheduled for 3:15 pm, i.e. after the clinical therapy blocks. However, for scheduling reasons, it was sometimes necessary for a participant, and sometimes for the therapist, to move the session to the morning. While seven participants indicated that the time of the session did not greatly matter, they preferred it not to be planned immediately before a meal. *"A meal after the session wasn’t doable—it invariably ended up in the bucket.”* It had been intended to spread the two ImRs sessions over the week, but circumstances did not always allow this. On the one hand this was experienced as pleasant for one participant, as she was then “done with it.” But on the other hand, she experienced it as very heavy. (Illustrative Quotes, Additional file 1: Table S3, point 5.2.4; Tips and Suggestions, Additional file 1: Table S4, point 4.5.)

### Themes emerged through the semi-structured interview

During the interviews, participants identified three other themes 1) hope/ perspective, 2) effect of ImRs on awareness, and 3) compassionate approach.

#### Hope/perspective

While five participants expressed they were feeling hopeless before the start of their trauma treatment (Additional file 1: Table S3; 1.1.3), they saw hope in the fact that research was being done into the possibility of treating trauma in underweight people. Three participants who had hoped that treatment might bring change were pleasantly surprised when their expectations were exceeded. *“Yes, I thought it might suit me, but I didn't think it would be as good as it was.”*

After the ImRs sessions, the five participants who had not dared to hope for a positive effect before the study seemed to have become more hopeful. They dared to look to the future: *“Yes, I'm very glad I did this. I had been at a loss, and thought, “yes, this is the only chance I have to get the combination [of ED and PTSD],’”* and *"I'm very glad it went this way, because […] if I’d done ImRs in a non-clinical setting, it would have gone really wrong*.” The participant who experienced no positive effects from the ImRs indicated that she was nonetheless glad she had been able to complete the 12 sessions. There had been no recurrence of the sense of failure that had followed her previous trauma treatment, when too many re-experiences had forced her to stop.

Afterwards, the three participants specified that it was helpful that the therapists had continued to hold out hope during this new treatment modality. In summary, it appears that everyone had been given hope by their experience of ImRs when underweight—some because they had succeeded in completing treatment, others because they noticed small changes, and others due to significant decreases in PTSD symptoms. (Illustrative Quotes, Additional file 1: Table S3, point 6.1; Tips and Suggestions, Additional file 1: Table S4, point 4.6).

#### Effect of ImRs on awareness

The interviews indicated that, in various areas, ImRs had initiated a process of awareness that had not previously been present. This had enabled six participants to re-frame the trauma: *“I was able to give a different colouring to what had taken place.”*

There are various examples of this awareness. One participant realized that she had PTSD. Others recognized the way things were connected, the severity of their trauma, the level of their tension, the lack of certain support figures, or their suppression of unpleasant memories. *"That whole piece of becoming aware of something that I've tucked away for so long under a block of concrete and landmines. It's… I compare it to a pimple that has burst. And now it's burst, and the pus can come out.”* In three participants, the awareness also triggered a grieving process. For two other participants, the awareness brought the hope of future improvement and insight. (Illustrative Quotes, Additional file 1: Table S3, point 6.2).

#### Compassionate approach

All the interviews testified to the importance the participants attached to a compassionate approach—something in which therapeutic attitude, group setting and ImRs method played a role.

One participant indicated that she had not had a good connect with her therapist, and thus changed therapists after two sessions. All other participants indicated that their therapeutic contact had been very compassionate. Comments included “Very nice,” “very pleasant,” “I felt taken seriously,” “she had expertise,” and “I felt she really listened to me.”

One participant indicated that therapists needed to realize that few such participants are securely attached individuals who have had only a single trauma. This makes it even more important for therapists to have a compassionate attitude*. "I'm just not used to it [getting help]: I always told myself I didn't deserve it. Then suddenly it's very strange that someone* does *help [during ImRs].”* Nine participants felt that the clinical group setting was also supportive, as compassionate support and understanding were both available. *“The group was very helpful, and really supportive and wished me well when I went to ImRs.”*

The ImRs method also contributed to not feeling alone. The therapist promoted this by stepping into the picture to help, which is experienced as both pleasant and compassionate. Because the therapist was constantly asking what someone needs, nine participants felt truly seen and understood, and saw the extent and reliability with which the therapist sympathized and helped. As a result, they saw this method as much less protocol-based than other, more standardized therapies such as EMDR. (Illustrative Quotes, Additional file 1: Table S3, point 6.3; Tips and Suggestions, Additional file 1: Table S4, point 4.5).

## Discussion

The clinical tradition to date has held that psychotherapeutic trauma-focused treatment should not be initiated in people who are underweight [13, 14]. The underlying assumption is that the conditions necessary for effective treatment cannot be met in such people, as cognitive functioning may be suppressed [15, 16, 17], not enough emotions are experienced [18] and emotion regulation is poor [19, 20]. The ability to experience emotions is important in trauma treatments. Olofsson et al. [37] showed that exposure to emotional and sensory trauma provided a good outcome.

However, the experience of participants in this study did not align with this presumption. They reported sufficient cognitive functioning, and also experienced a range of emotions. Despite their initial fears, they were also able to regulate these emotions, even though it was very scary to experience them again after such long periods of suppression. This emphasizes the importance of receiving caring support from group members, sociotherapists, and therapists. Other factors are equally important: to maintain or gain weight, to apply the imagining technique, to tolerate physical reactions, to be sufficiently open about the traumas they have experienced, a joint search for ways to remain in treatment and if necessary, to organize person-oriented adjustments by the therapist.

ImRs requires patients to use their imagination. Research showed that alexithymia (the inability to identify or describe one’s own feelings, externally oriented thinking) is common in ED and PTSD [38, 39, 40]. This difficulty to put words to feelings (or fantasize) may complicate the use of imagination. Future research should focus on whether there are limitations or modifications needed for this specific group with alexithymia to be treated with ImRs. In the interviews, three participants indicated two weeks after the ImRs sessions that they had not experienced any improvement with regard to the trauma symptoms. Neither, we note, did these participants experience a significant worsening of their PTSD and eating disorder symptoms. It is essential that the information given to future participants includes a warning about this range of possible responses.

Participants provided several tips for the use of ImRs in individuals with an ED during an underweight phase. One of the many tips participants provided is that unlike with other psychopathology groups, ImRs should not be planned immediately before a meal, and that the holding of the clinical setting is important with regard to maintaining the eating pattern. Inpatient admission helped with ImRs because there was extensive support immediately after the sessions to prevent participants from relapsing into disordered eating and weight loss after processing the trauma in the intervention. In line with Brewerton et al. [8] and Reyes-Rodriques et al. [3], it emerges that integrated treatment of ED and trauma is important. For follow-up research, it is important to take this aspect from extensive support during inpatient admission into account when considering trauma treatment for uED patients.

Straus et al. [41] defined compassion on the basis of five elements: “recognizing suffering, understanding the universality of human suffering, feeling for the person suffering, tolerating uncomfortable feelings, and motivation to act/acting to alleviate suffering”. Participants experienced ImRs as a highly suitable way of achieving these qualities, as the therapist is present in imagination at the traumatic event and can empathize with the situation very well. This is consistent with the findings of Bosch [42] and Olofsson [43] on the importance of the positive connection (with insecurely attached patients) with the therapist and getting encouragement for patients. Participants who had experienced EMDR indicated that ImRs is better suited to a compassionate approach. This may also explain why ImRs was completed by ten of the eleven participants who started it, the only dropout being one who experienced such positive effects (besides considerable fatigue) that she first wanted to work on her ED and then to continue the ImRs. For follow-up research, we recommend that a well-controlled comparative study is conducted between these two methods.

It is noteworthy that most participants specified that a process has been initiated which included a process of awareness—for some a grieving process—in which a process of increased self-compassion was combined with the feeling of not being left alone. These processes are well-known phases, and, for some participants, necessary steps on the road to recovery. Their great importance became apparent when it emerged that this group had felt very hopeless before the start of treatment. All had had a history of ineffective treatments followed by a long search for proper treatment; few had dared to have any expectations of ImRs. They reported that ImRs had given them hope again—thereby increasing their chance of better treatment outcomes [44, 45, 46] .

Our study showed that the therapists did not always address feelings of shame and negative body perception—that if a therapist did not ask about them explicitly, these issues were not discussed. However it is important to address the body [37]. To ensure that they are treated, and that therapists are alert to the participants’ and their own avoidance [47], it is important that any feelings of shame and negative body perception arising from the trauma are discussed explicitly. Conceivably, they were not discussed in our study because they were related to experiences that are not regarded as trauma in the DSM definition of PTSD. It might therefore be helpful if ImRs covered all early experiences associated with these feelings and not solely the trauma defined by the DSM. Possibly therapists do not discuss these issues because of uncertainties about how to integrate trauma treatment with an ED treatment, because of concerns about worsening ED symptoms, concerns that it would interfere with ED treatment or worsen self-harm [12]. Follow-up research should focus on the effects of ImRs in treating all early experiences associated with them and give attention to addressing existing concerns.

With regard to other effects, we found that the participants did not all experience decrease of anger, guilt, and PTSD symptoms two weeks after the end of ImRs treatment. This is in line with the multiple baseline study, which found a significant reduction in these variables only after three months [21]. As it is important to realize in this population that significant reductions in symptoms occur only after a few months, we recommend that participants in further research are interviewed after three months. While this interval may be necessary because participants need a little more time to work on the ED symptoms at the same time, it may also be that their underweight state is a contributing factor. And although it may be possible to start a certain process when underweight, a decrease in PTSD symptoms can be experienced only once the weight has normalized further. Follow-up research should establish whether ED symptoms (including BMI) are related to a decrease in PTSD symptoms after ImRs treatment.

### Implications

The results of our study imply that according to patients it is possible to treat PTSD with ImRs during an underweight phase in a clinical ED setting with adequate caring support, person-oriented adjustments and not schedule the trauma treatment immediately before a meal. The most important implication is that there are opportunities for this until now very difficult to treat group of patients.

The results of our study provides grounds for follow-up research such as a multi baseline case series study with more ImRs sessions or a well-controlled comparative study between EMDR and ImRs integrated with an ED treatment, in a caring and supportive setting. Several variants for an RCT are also recommended like an RCT of PTSD treatment in parallel compared with usual treatment of just the ED, or parallel vs sequential treatment of the ED and the PTSD. It is important to add a cost-effectiveness study, to take into account the experienced compassion in relation to drop out numbers and effectiveness of the treatment, and to include the diagnosis of alexithymia. Furthermore, we recommended follow-up measurements after three months and 1 year and establish whether ED symptoms (including BMI) are related to a decrease in PTSD symptoms after trauma treatment. For follow-up research in outpatient ED settings, it is essential to organize extensive support for care after the trauma treatment sessions and support at meals.

### Strengths and limitations of this study

Although the researcher (i.e. the first author) is well versed in the treatments and can interpret the experiences participants have during therapy, there is a risk that her professional view may have led her to attribute meanings to the participants’ experiences. The participation of the second researcher was intended to rectify this by providing experiential expertise and safeguarding the participants’ perspectives in the analysis and the interpretations were checked with the participants who thought they were accurate. Another limitation is that the interviews were conducted two weeks after the end of the ImRs, and thus provided no insight into the participants' opinions and experiences in the longer term. Neither was the timing of the interview linked to the decrease in ED symptoms. Finally, since this was a single case series study, the number of interviews was limited.

## Conclusion

Our study aimed to improve the trauma treatment and to test the feasibility of trauma treatment provided to people with an ED who, while still underweight, underwent ImRs during clinical treatment for their ED. A second aim was to understand these participants’ experiences of ImRs and their perspectives on it.

Our results show that, thanks to the opportunity to have simultaneous treatment of their ED and PTSD in the safe environment of the clinic, participants dared to have trauma treatment despite the feelings of hopelessness that had remained after their experiences of earlier treatment. An important component of daring to feel and daring to continue eating despite the fear of the emotions they might feel was the support and compassion of peers and the clinical team. Even though they had been underweight, participants indicated that they had been able to undergo treatment because their ability to concentrate, and to experience and regulate emotions, had all been sufficient.

The study also demonstrates the importance of properly establishing in advance and during the ImRs treatment what each participant will need to complete trauma treatment. Such preparation and adjustment contributed to their experience of ImRs as a pleasant, compassionate method, in which their sense of being on their own had been reduced by the therapist’s empathy and personal focus. Two weeks after the ImRs treatment, participants also indicated that they had gained more hope about processing their trauma and ED symptoms. With regard to the long run, however, they were uncertain whether they could expect more effect from the ImRs than from other treatments. It is essential that the information given to future participants includes an explanation of possible delayed effects.

To better tailor treatment to participants’ needs, ImRs should not be scheduled directly before a meal. However, shame and negative body experiences should be elicited explicitly, and everything an individual needs to sustain treatment should be carefully adjusted and monitored.

## Supplementary Information

**Additional file 1.** **Table S1.** Interviewees’ demographic and clinical data.

## Data Availability

Reasonable requests for data will be considered by the authors under the condition that the European General Data Protection Regulation (GSPR) is guaranteed for these sensitive participant data, and an appropriate analytic plan is included.

## Footnotes

1 National Centre for Social Research. https://www.natcen.ac.uk/. Accessed 27 Feb 2022.
