# Supplementary material for: Perspectives of underweight people with eating disorders on receiving Imagery Rescripting trauma treatment: a qualitative study of their experiences
Source: J Eat Disord. 2022 Nov 30;10:188. doi: 10.1186/s40337-022-00712-9 (PMC9710063; doi:10.1186/s40337-022-00712-9)
Supplement: Supplementary file 1 — Additional file 1. Table S1. Interviewees’ demographic and clinical data. [file 40337_2022_712_MOESM1_ESM.docx]

| ***Variable*** | | |
| --- | --- | --- |
| ***Age (Mean, SD)*** | *Range 16-58 (n=12)** | *25.7       (10.5)* |
| ***Gender (Number, %)*** | *Female* | *12          (100%)* |
| ***Educational level completed (Number, %)*** | *Pre-vocational secondary education* | *3            (25%)* |
|  | *Secondary vocational education* | *5            (41.6%)* |
|  | *Senior general secondary education* | *1            (8.3%)* |
|  | *Pre-university education* | *2            (16.7%)* |
|  | *University education* | *1            (8.3%)* |
| ***Feeding and eating disorder (Number, %)*** | *Anorexia Nervosa* | *10          (83,3%)* |
|  | *Other specified feeding and eating disorder* | *2            (16.7%)* |
| ***Body Mass Index at start of study (Mean, SD)*** | *Range 14.9-17.8*** | *15.6       (1.2)* |
| ***Body Mass Index at start of ImRs phase (Mean, SD)*** | *Range 14.6-18.4*** | *16.7       (1)* |
| ***Number of participants per trauma category (LEC-5 categories***)*** | *Physical assault (for example, being attacked, hit, slapped, kicked or beaten up)* | *5* |
|  | *Sexual assault (rape, attempted rape, made to perform any type of sexual act through force or threat of harm)* | *6* |
|  | *Other unwanted or uncomfortable sexual experience* | *2* |
|  | *Combat or exposure to a war zone (in the military or as a civilian)* | *1* |
|  | *Any other very stressful event or experience* | *1* |
| ***Previous trauma treatment*** | *EMDR* | *4* |
|  | *Other* | *2* |
|  | *None* | *4* |
| ***Total CAPS-5**** score  (Mean, SD)*** | *Range Caps total 33-58* | *46.3      (9.2)* |

**Table S1**

*Interviewees’ Demographic and Clinical Data (N=12).*

* One outlier aged 58. Age range without outlier 16-30, Mean 22.5, SD 4.7

** Without the dropout and the participant who did not start ImRs

*** Weathers, Blake, Schnurr, Kaloupek, Marx, & Keane, (2013).

****Clinical-Administered PTSD scale for DSM-5.

**Table S2**

*Client Interview Topic List*

| *Subject* | *Theme, statement or question* | *Interviewer’s checklist* |
| --- | --- | --- |
| 1. *Introduction* | *Information about the interview* | ***The interviewer should:***  *Introduce herself* |
|  |  | *Explain the research* |
|  |  | *Indicate the duration of the interview* |
|  |  | *State the purpose of the interview* |
|  |  | *Ask the client’s permission to record the interview* |
|  |  | *Open the interview* |
|  |  | *Indicate that it’s fine if the client doesn’t want to answer a question. All he/she has to do is make, and asks him/her to make this known* |
|  |  | *Explain that the record will be anonymous* |
|  |  | *Explain that data will be destroyed within 5 years* |
|  |  | *Ask permission to do a "Member check” (i.e. feedback analysis as a check for validity)* |
| 1. *Sociodemographic* | *“Before we start the interview, I would like to check some sociodemographic items.”* | *Ask about the client’s diagnosis, age, BMI, gender, completed education, socioeconomic status, training and work* |
|  |  | *Ask the client to list any previous therapies related to childhood traumatic experiences and ED* |
| 1. *Experience* | *“What was your experience of ImRs during underweight?”*  *“What was your experience of the imagery exercises?”*  *“What was your experience of your therapist?”* |  |
|  |  |  |
|  |  | ***The interviewer should ask:***  *About the client’s contact with the therapist* |
|  |  | *About the quality of the attention the therapist paid to the client* |
|  |  | *About the therapist’s approach to the client. Did the client feel that he/she was taken seriously?* |
|  |  | *About the quality of communication between therapist and client* |
|  |  | *About the therapist’s expertise* |
| 1. *Experiencing emotions* | *“I’d like to ask you about your experience of feeling emotions during ImRs treatment.”* | *Whether the client felt any emotions during ImRs* |
|  |  | *Whether the client’s emotions changed during the treatment* |
| 1. *ED affecting trauma and vice versa* | *“I’d like to ask some questions on the combination of trauma and eating disorder.”* | *Whether the client’s trauma affected his/her ED treatment* |
|  |  | *About the relationship between the ED disorder and the trauma.* |
|  |  | *Whether this relationship changed during ImRs* |
| 1. *Participation and expectations regarding the ImRs therapy* | *“I’d like to ask you about your expectations of ImRs before you started it.”* |  |
|  | *“I’d like to ask why decided to have the ImRs therapy.”* | *Whether the information about the ImRs before it started was clear, complete, and useful.*  *Whether anything was missing from this information.* |
| 1. *Experiences of clients with other forms of therapy* | *“I’d like to ask whether you’ve had any previous experience of treatment.”* | *Whether the client’s perceptions, feelings and experiences differed between ImRs and those he/she experienced in other forms of trauma treatment.* |
|  | *“Can you tell me more about that?”* |  |
| 1. *Opinions on ImRs* | *“What are your ideas and opinions on treating trauma when you’re underweight?”* |  |
|  | *“Do you think that your treatment was affected in any way by your being underweight? For example, did it hinder it or help it?”* |  |
|  | *“In your opinion, how effective is this ImRs method?”* |  |
|  | *“Did you and your therapist use various different interventions during ImRs? If not, please explain as necessary.”* | *Whether any interventions should be added or removed.*  *Whether there had been trouble with certain aspects of the therapy.* |
|  | *“I’d like to ask your opinion of these different interventions.”* |  |
| 1. *Effect of ImRs* | *“What do you think the effect of ImRs will be in the longer term, weeks and months from now?* |  |
| 1. *Other* | *“We’re almost at the end of the interview. Are there any items that haven’t been discussed?”* |  |
|  | *“Is there anything you would like to ask me?”* |  |
|  | *“Thank you very much for participating.”* |  |
| 1. *Additional questions** | - - *“How great was your belief in the ImRs technique before you started?”*   - *“How did the imagining go?”*   - *“Could you feel emotions during the sessions?”*   - *“Did the trauma treatment affect your eating?”*   - *“Did it affect you that someone was trying to meet your needs?”*   - *“What was your experience of the second six sessions, in which you had to rescript yourself?”*   - *“Were any special things or images used in the rescripting?”*   - *“Did your weight influence the trauma treatment?”*   - *“Was it confronting to work on the lack of needs you felt?] after your trauma period?”*   - *“Do you now consciously try to use the imagery technique yourself?”*   - *“How did you feel after the ImRs sessions?”*   - *“Was the support and care of the clinical treatment team important to you?*   - *“Was the trauma still current when you started ImRs?*   - *“Could you determine how the ImRs went?”*   - *“How was that for you?”*   - *“What do you think of the number of sessions (12), Should there be more but shorter sessions?”* | |

** These questions were added as a result of the first three interviews.*

**Table S3**

*Themes, Subthemes and Selected Illustrating Quotes*

| Themes | Sub-themes | Sub-theme categories | Quotes |
| --- | --- | --- | --- |
| 1. Expectations of IrMs | 1.1 Prior expectations | 1.1.1 Positive | "I was very happy there was a clinic somewhere that was willing to offer trauma treatment despite my being underweight." |
|  |  | 1.1.2 No expectations | "I didn't really have any particular expectations. I thought, ‘I'll just let it come to me.’" |
|  |  | 1.1.3 Not daring to hope | "I didn't even dare to expect things to get better, as I’d been through trauma treatment before and none of it had worked. And I thought, ‘well, if I expect something from this again, I'll be so disappointed yet again if it doesn’t work …’ And so I was, like, well..., I won't expect anything.” |
|  |  | 1.1.4 Difficult to get a good idea of ImRs | "I’d imagined it beforehand, but it was a bit more intense than I’d thought.” |
|  |  | 1.1.5 Doubt | "A therapy like that isn’t going to help me, is it?” |
|  | 1.2 Preparation | 1.2.1 Information | “I’d received a whole paper with the explanation, and she also explained it to me on the spot.” |
| 2. Ability to participate in ImRs | 2.1 Ability to control attention and to concentrate during ImRs | 2.1.1 Sufficient ability | “Yes, I was generally able to concentrate sufficiently.” |
|  |  | 2.1.2 Stable somatic situation | “This treatment shouldn’t have been offered when I didn't eat anything at all, because then I felt quite little and had no concentration at all. And my blood sugar was also very low.” |
|  |  | 2.1.3 Ninety minutes | “I think if the sessions were a little shorter it might be more effective.” |
|  |  | 2.1.4 Extraneous noises | "The space needs to provide very few stimuli, as [stimuli] can break your concentration and push you into a reliving situation. At one point I heard all kinds of different voices of people in the hallway, and that didn't go well either....” |
|  | 2.2 Ability to feel | 2.2.1 Sufficient opportunity to feel | “I actually experienced it really intensely – yes, very intensely. Very often I also felt the reactions in my body, so to speak. So yes, I really did experience it.” |
|  |  | 2.2.2 Avoiding feeling | “I noticed that I pushed away the real emotions a bit. But in terms of tension: the therapist said that she could see when the tension got high. Yes, I found it difficult to feel emotions, so I pushed them away.” |
|  |  | 2.2.3 Ability to feel during weight gain | "No, I don't really feel that my ability to feel changed very much.”  “The intensity of the emotions didn’t change – before, during or after gaining weight.” |
|  |  | 2.2.4 Being able to feel better in the second six sessions | "From the moment I started to intervene, it became a bit easier.” |
|  | 2.3 Ability to regulate emotions | 2.3.1 Scary; getting emotions and lingering in them | "I'm not used to feeling emotions at all any more, I've always pushed that away, and then it's just scary when it's there again.” |
|  |  | 2.3.2 Managing not to get caught up in the emotions | "And then I was crying really hard, and then it was okay, too. Not that I got stuck in it, because that’s what I was really afraid of – getting stuck in it." |
|  | 2.4 Ability to gain weight: role of weight or underweight | 2.4.1 Difficult to gain weight, but possible | "Even though I felt very emotional, I was largely able to follow the growth line and keep to the eating plan. It didn't always work out perfectly, but I hadn’t expected it to.” |
|  |  | 2.4.2 Targets too high | “Eating became extremely difficult because of all the re-experiences. It didn't get any easier, but they helped me look at ways of ensuring I’d still get enough, even if eating was such an effort. If I hadn't been in that environment, I think I’d just have stopped eating. But now we could at least work on making sure that I didn’t lose more weight. So yes, I did experience that as positive, but no, gaining a kilo per week at the time of the ImRs was out of the question.” |
|  |  | 2.4.3 Not possible to gain weight | “Well at least I didn't gain weight during the period of ImRs.” |
|  | 2.5 Ability to imagine | 2.5.1 Easy | “At the first sessions, when we started, I was amazed how well I could imagine it. Amazed that I took the therapist along in the story quite well, and that she then intervened – I could see all of that very well. I was a bit overwhelmed by what I actually remembered from back then, and also just by the feeling that I could feel everything I’d felt back at the time. It touched me a lot that someone stood up for me in that situation.” |
|  |  | 2.5.2 Had to get used to it | “In the first session I did have a hard time with it, but after that it wasn’t difficult.” |
|  |  | 2.5.3 The therapist or herself in the image did not work | “It just didn’t work out. I guess I just had in my head that, yeah, it wouldn’t go that way. So I guess that that just blocked me the whole time. Also with myself in the picture.” |
|  |  | 2.5.4 Ability to fantasize | "No, because I’m a bit rationally minded, I get very uncomfortable, like it's very weird what I'm doing.” |
|  |  | 2.5.5 Difficult | “Imaging it, then really keeping my attention and really getting into that feeling – I found it really difficult. Because I’m constantly very aware that I’m not in that situation, and that there’s someone there who I don’t know at all. Yes, I find that difficult....” |
|  | 2.6 Ability to engage in ImRs due to its physical effects | 2.6.1 Physical reactions | “Sweating, my breathing, picking at my skin, my heart beating...." |
|  |  | 2.6.2 Physical reactions decreased | “My body reacts to it less intensely. I’m also better at telling myself ‘it's not now, it was then.' And then I try to see the rescripted scene in front of me – that it ends well, etc.” |
|  | 2.7 Ability to be open about the trauma | 2.7.1 Topicality of the trauma | “I really thought the perpetrators were here, because they'd sent me a message letting me know where they were, which really, really shocked me.” |
|  |  | 2.7.2 Difficulty of being open | “I've now written everything out once, but just so the therapist would know what had happened, as I find it very difficult to talk about it.” |
| 3. Perceived effect of ImRs | 3.1 Perceived effect on anger | 3.1.1 Difficulty showing anger | “Yes, I wanted to be angry too, but I didn't dare to show it. Because then all you get is this sweet little voice, that’s very angry, you see?” |
|  |  | 3.1.2 Anger and less anger | “I was very angry at what had happened – angry because, for example, no one ever saw anything or did anything to stop it.”  “And the feeling of anger, it’s also changed. I think it’s decreased.” |
|  | 3.2 Perceived effect on guilt | 3.3.1 Less guilt and more guilt | “I now have less of an idea or belief that it was my own fault. I still can't quite believe it, but it’s less now.”  “Because I felt even more guilty about it, it started playing in my head even more, and I suffered from it even more, also unconsciously.” |
|  |  | 3.3.2 Meaning of the trauma changed | “Together with the therapist, I could give a different meaning to the situation than the one I’d given before. And, as it were, I could colour the situation in a way that made it clear that what had happened just wasn't permissible and that something very different should have happened – something I’d actually deserved. So, in that sense, the attention has been diverted from the shame and the feelings of guilt, and there’s now a much greater focus on what I actually needed. That’s what I find really beautiful – that in this way the situation has sort of been given a different theme.” |
|  |  | 3.3.3 No difference | “It came out in the rescripting that it wasn't my fault, so we left the situation. But while I could imagine this really well, that just wasn't the way it had happened in real life." |
|  | 3.3 Perceived effect on eating | 3.4.1 Eating now more difficult | “Because of the ImRs, it’s now more difficult with food. For example, I can’t take food whose colour is white.” |
|  |  | 3.4.2 ED as a distraction from trauma | “I definitely think my eating disorder helped a lot to push everything away. And... I think it still does.” |
|  | 3.4 Generalisation of perceived effects and participants' ability to apply ImRs themselves | 3.5.1 Have not yet internalized the method | “Sometimes I try to use an Imagination very consciously, but it’s all very mixed up and... but, well, if I’m in a real re-experience, or if I dissociate completely, there’s nothing […] of the Imagination left in it at all.” |
|  |  | 3.5.2 Unsure whether any effect of the ImRs can be expected over time. | “I have no idea what to expect. Regarding improvement, effects ... I don’t know.” |
|  |  | 3.5.3 Making some independent use of the ImRs method | “Yes, I really try to stop the image consciously and start the new one.” |
|  | 3.5 Perceived effect on negative body perception | 3.6.1 Was addressed, but had no effect | “We did talk about [body perception], saying among other things that I’m not dirty and that I can't be fat with such a BMI. But I haven't had any therapy on that, so I don't find it very strange [that it doesn't feel the way I’d like it to be]. ” |
|  |  | 3.6.2 Linking the negative body awareness to the ED | “There was also a questionnaire with – that I felt 'too much,' or that I felt 'disgusted with myself,' or that I felt 'too fat'. Well, that's actually still there. But I think that's purely because of the eating disorder. It couldn't really be reduced by this research.” |
|  | 3.6 Perceived effect on PTSD symptoms | 3.7.1 Positive effects | “I can stay with it better – I don't get away from reality as much. There was a period last year when I got away from reality at least once a day. Now I can recognize better when I'm being triggered, so I start doing something with it to avoid a re-experience or getting away from reality.” |
|  |  | 3.7.2 Negative effects | “It was very heavy. I really had the feeling that everything was being racked up and that it was being rewritten, as it were. As the original images were being brought back up, they came to the fore again. And in these re-experiences I didn’t manage to get back to what was done in the rescripting. So that caused trouble and made it very intense.” |
|  | 3.7 Perceived effect on grief process | 8.8.1 Mourning process | “I just felt very much what I’d lacked so much back then. If only someone had helped me. It was very intense. I thought it was very bad.” |
|  | 3.8 Perceived effect on shame | 3.9.1 Shame about one’s own fantasies | “Even though I could do anything I wanted to the perpetrator, there was still a bit of shame there when I thought what I’d want to do to him.” |
|  | 3.9 Perceived effect on self-compassion | 3.10.1 Self-compassion increased | "But I do think it's beautiful that things that are actually so poignant could be rewritten in a loving way.” |
|  |  | 3.10.2 Self-compassion did not increased | I do understand of course that you can't brush away the past, but I don't have the idea yet that it's really...uhh, that it's cleared up and has a place where I can look at it with more compassion or anything like that. As for comments like “it's not your fault,” etc, well, you can say that now, but that’s not the way I feel.” |
|  | 3.10 ImRs and its hypothetical future effects | 3.11.1 Don’t know | “As far as improvement and effects are concerned... I don't know.” |
|  |  | 3.11.2 Doubt about effects | “But I notice that it doesn't change very much in my mind. So then I think... why would it suddenly go or change?” |
|  |  | 3.11.3 Thinking effects may be likely | “I can imagine for example that, now, in my imagination, I’d be able to say to someone what I wouldn’t have been able to say otherwise. That perhaps it can have an effect, yes.” |
|  |  | 3.11.4 Nice start | “I think this is a very nice one to start with, so to speak, that its effect keeps trickling along. So maybe it's not quite enough to really, completely get those beliefs. It hasn’t completely gone or completely changed, but, yes, I do think it's a nice start.” |
| 4. Experience of ImRs | 4.1 In general | 4.1.1 Went well | “It's nice that I could imagine it and change it myself.” |
|  |  | 4.1.2 Control | “What helped me the most was that X said I was the boss, that I got to decide what was going to happen.” |
|  |  | 4.1.3 Avoidance | “I’m inclined to push it away, and we haven’t gone much further than the place I normally push it away to.” |
|  | 4.2 First six sessions | 4.2.1 Modelling/re-parenting | “In the first six sessions I liked the fact that the therapist intervened.” |
|  |  | 4.2.2 Needs | “I, uh, couldn't think of anything, so usually the therapist would come up with suggestions and I could choose from them. Choose what I thought I’d need most, but could I figure it out myself? No. That did change over time, though. As the sessions progressed, I got better and better at identifying what I needed.” |
|  |  | 4.2.3 Realism of script | "But when we built a shield around the house together at a hotspot in the image, I was, like, 'that's just not possible.” |
|  | 4.3 Second six sessions | 4.3.1 Pleasant | “It went better than expected, which is why I was also able to get into that feeling more. And I was also able to imagine that it *can* work like that, and that you’ll also really notice a difference.” |
|  |  | 4.3.2 More difficult | “I had a lot of trouble with the idea that I was going to intervene as an adult person, because I don't feel like a strong healthy adult. As a little girl, I found it very difficult to go and help myself. I was like, ‘but I’m no good to you at all, as I’m absolutely nothing.’ I had that a lot, and it caused me a lot of trouble.” |
|  |  | 4.3.3 Finalize imagination | “Because I was completely in the image, I was really a bit lost in thought. I felt a bit between the real world and the imaginary one.” |
| 5. Conditions under which ImRs is given | 5.1 Clinical setting | 5.1.1 Support required, nice | "The contacts and moments of moments with other group-mates help to distract me from unpleasant feelings. The support and help from the sociotherapists also help me feel I’m not alone.”  “I liked the fact that it was in the clinic. If I’d had to go home afterwards, that would have been too hard for me.” |
|  |  | 5.1.1. 2 Needed more support | " I did have a situation when I was by myself here on the unit and couldn't find anyone. I’d then gone to my room and had a lot of panic. Finally I pressed the button, and I think X ended up being with me.” |
|  |  | 5.1.1.3 Preferred to be alone | “I found it very nice that I could go home and do my own thing instead of being forced to do something with other people from the group, or to go and sit when I didn't feel like it at all, when someone might ask again how it went. Though that’s very sweet, at a time like that [after ImRs] I didn't feel like it. So I actually liked the fact that I could just go home and do my own thing.” |
|  |  | 5.1.2 Many participants at the same time | “If there's someone else in there [in the clinic with PTSD] it's kind of tricky because they can trigger each other.” |
|  |  | 5.1.3 Belief about ImRs | "I’d seen other people who came out very heavy, so I really doubted that I’d come out of it quite light-hearted. But with me it wasn’t so bad. So I thought to myself that I didn't really deserve someone asking me how it had gone. I had a hard time dealing with that.” |
|  |  | 5.1.4 Needed support with meals | “Eating became extremely difficult because of all the re-experiences. It didn't get any easier, but even if eating was so difficult then, [the sociotherapists] were doing everything they could to see how we could make sure that I still got enough [food] in. If I hadn't been in that environment, I think I’d have just stopped eating.” |
|  | 5.2 ImRs treatment | 5.2.1 Clinical setting in combination with ImRs | “The combination of all-day therapy and trauma treatment was just too much.” |
|  |  | 5.2.2 Number of ImRs sessions | “What’s needed per person really varies a bit, as everyone needs something different, of course. One person has more and another person has less, so... For example, I have a lot of different things [traumas], actually… and another person might have only one thing [trauma]. So I think that also makes a difference...” |
|  |  | 5.2.3 Timing of ImRs | “I guess it was good timing” |
|  |  | 5.2.4 Scheduling of ImRs | “Uhm, I’ve been feeling tired for a while. That may also have had to do with being underweight – really very tired. I felt really tired at a quarter past three, after such a day here. So I actually needed rest, and most sessions were scheduled at exactly a quarter past three. So I couldn’t rest for a moment and then had to go right on.” |
|  |  | 5.2.5 Therapeutic attitude | “But I really enjoyed being with her, because she talked so calmly, and because she wanted to help me in that picture. I really enjoyed it.” |
| 6. In-depth analysis | 6.1. Hope/ perspective | Better than expected | “I have a bit of trouble accepting help, so I always think it will help others but not me. But now I’ve found out that it will also help me.” |
|  |  | Developed more hope | “Now I have the idea that this is just the start, and that's good. And that there are now people who at least know what they’re doing, trying to help me tackle the eating disorder and find out what's behind it.” |
|  |  | No effect but no failure | “Yes, I did finish it completely, yes... Only I don't feel like it helped me very much or anything.” |
|  | 6.2. Effect on awareness | Realising you have an ED | “I myself didn’t realize that I really had an eating disorder. It was only when I started reading about it that I realized that it may really be the case.” |
|  |  | Realising you have PTSD | “Because, uhm, I didn't know at all that I was suffering from PTSD or something.” |
|  |  | Suppression | “Yes, I’ve become aware that I have indeed missed some things very much, or that certain people haven’t given me things I needed.” |
|  |  | Grieving | “The awareness has become so strong that it actually caused me a great deal of grief.” |
|  | 6.3. Compassionate approach | Therapeutic attitude | “I really enjoyed talking to her, because she talked so calmly, and because she wanted to help me in that picture. I really enjoyed it.”  “The warmth, confidence, empathy, thinking along with me and being innovative about how to keep me present. […] I’m super happy how she dealt with things. Outstanding!” |
|  |  | Attachment | “I still feel quite cut off from the child who needed something other than what she got.” |
|  |  | Compassion in the group setting | “And here [in the clinical setting] ... they do everything they can to help me. I’ve never had anything like that happen to me before.” |
|  |  | Compassion in the ImRs method | “I certainly always felt as if I was being listened to a lot more [than in other therapies]. Actually being understood much more.” |

**Table S4**

*Participants’ Tips and Suggestions*

| **Tips** | | |
| --- | --- | --- |
| 4.1. In advance | | |
|  | | **Therapists should do the following:**   - Check very carefully that participants are safe and that their traumas have stopped. - Help to break any secrets surrounding trauma by involving parents, family or close friends if possible. - Explain ImRs well, stating that it may be a tough experience, and also stating clearly that each participant is likely to react to it differently. - Give participants the opportunity to write down the whole story if it is difficult for them to talk about their trauma. - Explain ImRs on paper and also during the rescripting trial in the first session.   **Overall:**   - ImRs should be advertised more. “Currently, people know only about EMDR and Imaginary Exposure.” |
| 4.2. Participants’ability to: | | |
| - Concentrate | | - The sessions should be shorter or be scheduled more in the morning than in the afternoon. - There should be opportunities for listening to the recorded sessions. - A low-stimulus environment is important to good concentration. |
| - Feel | | - The ImRs should be offered in the clinic, where there is a safe environment. This would support learning how to regulate emotions and daring to allow them. |
| - Regulate emotions | | - If it enables someone to experience more emotions, rescripting should be more realistically. |
| - Weight growth | | - During the part of the programme with the ImRs sessions, the weight-increase line should be applied more flexibly – 300 grams rather than 700 grams, for example. |
| - Imagery | | - If the options for imagination are limited in any way, participants and therapists together should make a list of subjects that can be used in the rescripting. - If someone is unsure what a need might be, suggestions should be made for needs that could be tried out in rescripting. It should then be checked whether this works. - The need to tell the whole story of the trauma is also a need. It’s possible to listen to the whole story very carefully in rescripting, especially if someone has never previously disclosed the trauma. |
| - Body effect | | - For limb failure, it helps to do the ImRs standing up and to do the ImRs in an area where the participant can stay after the session. - For severe physical reactions to ImRs, the two weekly sessions should be spread over the week. |
| - Openness | | - If it is difficult to talk about traumas, the opportunity should be given to write the events down. |
| 4.3. Effect of ImRs on: | | |
| - Shame | | - Explicit questions should be asked about feelings of shame. - If there is shame, a participant should be allowed to do in their thoughts whatever they wanted to do. They do not need to speak out loud. - If a questionnaire is used that asks about shame, this should be discussed explicitly in the session. |
| - Negative body perception | | - Specific attention should be given to negative body perception. - An additional ImRs session should be organized about the body. |
| 4.4. ImRs techniques | | |
| - Imagery (dissociation, re-experiences) | | - Per individual, it should be clearly agreed how many traumas are going to be rescripted. - The option should also be offered of occasionally allowing an event to return several times in order to be completed. - Working on one event per session rather than several should be considered.   To stay in the here and now with dissociation and re-experiences:   - A cord should be used to connect therapist and participant. This can then be pulled to keep the participant in the here and now. - A scented bag should be used. This can then be sniffed at to keep the participant in the here and now. - Participants should continually scan the environment with all their senses. - The ImRs should be done standing up. |
| - First 6 sessions | | - Therapists should have confidence in the technique. - When prescribing rescripting in the first 6 sessions, the therapist should act as a model. |
| - Second 6 sessions | | - If someone needs more time to get used to "stepping into the picture themselves," options should be provided for scheduling additional sessions. |
| - Ending ImRs session | | - Therapists should check the way in which participants leave the room, and whether they need assistance. - If necessary, a therapist should accompany a participant on the way to the sociotherapists. - After the ImRs, participants should be given the opportunity of talking to a sociotherapist. - To ease the transition from the rescripting to the here and now, the light in the room should be turned off. - If someone is unable to walk, they should be provided with a wheelchair. |
| 4.5. Contextual conditions | | |
| - Clinical setting | | - A safe environment should be provided in which it is safe to feel and express emotions. - Options should be offered for participants to talk with group members, roommates and sociotherapists. - Participants should be offered the opportunity to go for a walk alone or under supervision. - It should be ensured that the therapy rooms are low in stimuli - Therapists should not underestimate how difficult it is to ask for support. - Because participants’ experiences of ImRs differ, the clinical treatment team should try not to form general ideas about how difficult it is to undergo it. |
| - Multiple participants | | - It should be carefully considered whether providing trauma treatment to several participants simultaneously will be supportive or likely to trigger trauma symptoms. - Ensure adequate staffing with multiple participants at the same time |
| - Mealtime support | | - To keep participants eating during the ImRs period, therapists be aware of the importance of providing them with support at mealtimes. |
| - Therapeutic attitude | | - Therapists should not panic. Together with participants, they should look for ways of making the ImRs more pleasant. |
| - Number and duration of sessions | | - If there is the option to stop earlier, 90-minute sessions are fine. This will enable participants not to feel guilty if they don’t stick with it. - If it is decided to make the sessions shorter, a higher number of sessions should be offered over a period of more than 6 weeks.. - If possible, it should be agreed how many sessions are needed per individual. |
| - Time and timing | | - ImRs should not be scheduled immediately before a meal, especially a hot meal. - Participants should be advised to seek some distraction (i.e. stimulation of all the senses) before the meal starts. - The sessions should be spread over the week. - ImRs should be started only after a settling period in the clinic. - It should be agreed per person when it is good to start ImRs. If possible, participants themselves should be given control over this. |
| - Research | | - Because more specific questions are much easier to answer, questionnaires should, if possible, ask about the trauma that has just been dealt with in the session. - If recordings were made or if no trauma treatment will be provided for a period of time after the ImRs treatment, this should be clearly communicated. - It would be nice if it were clear in advance when the ImRs would start. - In people with an eating disorder, care should be taken about expressing exclusion criteria in terms of BMI. |
| 4.6. Other | | |
| - Hope/ perspective | - It was helpful that the therapists held out hope during this new treatment modality. | |

**Box S1**

*Twelve-Session Imagery-Rescripting Protocol*

| General | - Twelve individual 90-minute ImRs sessions - Offered over 6 consecutive weeks. |
| --- | --- |
| Aim | - To change the meaning of traumatic experiences by participating in imagined interventions that correct the dysfunctional emotional and interpersonal meanings attached to the trauma. |
| Brief summary of the rescripting procedure | - The trauma memory is activated, i.e. the patient imagines the start of the traumatic memory. - When there is enough emotional activation, the rescripting starts. - The therapist, and later the participant (each from their current perspective), then rescripts the traumatic experience to provide a more pleasant outcome, while imagining this new script in the liveliest way possible. |
| Intended results | - To change maladaptive beliefs, to develop more control over images, and to improve the options for reassuring oneself (Long & Quewillon, 2009). |
| Advantage | - The patient does not have to relive the full trauma in all its details |
| Warning | - As the rescripted outcome contains new and unexpected information for the patient, a lasting change of memory is created (Finnie & Nader, 2012). |
| First session | **In this preparatory session:**   - a therapeutic alliance is formed, - the treatment and its rationale are explained - a list is established of the traumas a participant has experienced - the order is discussed in which participant and therapist will address the traumas - The participant’s current living circumstances are checked in order to verify whether there is enough distance from the perpetrator and enough safety to conduct trauma processing - to familiarize the patient with the ImRs technique, a mildly negative childhood memory is used in a pilot ImRs - the session is evaluated - Homework: to read the explanatory ImRs hand-outs and to reread the list of trauma themes (to change items and/or their order if necessary before the following ImRs session). |
| Sessions 2-12 | **In these sessions:**   - The therapist checks for any intrusions, nightmares, and emotions that followed the previous session - Patient and therapist then discuss how the previous session affected the patient - They then agree which trauma theme to start the session with - The participants closes his/her eyes and retrieves the traumatic memory - The therapist (session 1-6) or participant-as-adult (session 7-12) steps into the image - The therapist or participant-as-adult intervenes, and the participants imagines this intervention - The therapist checks whether the imagined situation is effectively under control and meets the child’s needs. - If intervening was not successful, they rewind and start again - The session ends when the participant-as-child says “It’s OK.” - The rescripting is evaluated - If time allows, another memory is rescripted - The session is evaluated - Homework is assigned: to review the trauma list with regard to its order and the needs or wishes for addressing traumas.   For a more detailed treatment description, see Raabe et al. (2015). |
